# Effects of melatonin and human follicular fluid supplementation of in vitro maturation medium on mouse vitrified germinal vesicle oocytes: A laboratory study

**DOI:** 10.18502/ijrm.v19i10.9821

**Published:** 2021-11-04

**Authors:** Razieh Doroudi, Zohre Changizi, Seyed Noureddin Nematollahi-Mahani

**Affiliations:** ^1^Department of Anatomy, Afzalipour School of Medicine, Kerman University of Medical Sciences, Kerman, Iran.; ^2^Leishmaniasis Research Center, Kerman University of Medical Sciences, Kerman, Iran.; ^3^Kerman Physiology Research Center, Institute of Neuropharmacology, Kerman University of Medical Sciences, Kerman, Iran.

**Keywords:** Vitrification, Germinal vesicle, In vitro oocyte maturation, Melatonin, Follicular fluid.

## Abstract

**Background:**

Vitrification as the most efficient method of cryopreservation, enables successful storage of oocytes for couples who undergo specific procedures including surgery and chemotherapy. However, the efficacy of in vitro maturation (IVM) methods with vitrified germinal vesicle (GV) oocytes could be improved.

**Objective:**

As melatonin and follicular fluid (FF) might enhance IVM conditions, we used these supplements to assess the maturation rate of vitrified GV oocytes and their artificial fertilization rate.

**Materials and Methods:**

Four hundred mouse GV oocytes were harvested, vitrified, and assigned into control (C-Vit-GV) and treatment groups of melatonin (M-Vit-GV), human follicular fluid (HFF-Vit-GV), and a combination (M + HFF-Vit-GV). A non-vitrified group of GV oocytes (non-Vit-GV) and a group of in vivo matured metaphase II (Vivo-MII) oocytes served as control groups to evaluate the vitrification and IVM conditions, respectively. Maturation of GV oocytes to MII and further development to two-cell-stage embryos were determined in the different groups.

**Results:**

Development to two-cell embryos was comparable between the Vivo-MII and non-Vit-GV groups. IVM and in vitro fertilization (IVF) results in the non-Vit-GV group were also comparable with the C-Vit-GV oocytes. In addition, the IVM and IVF outcomes were similar across the different treatment groups including the M-Vit-GV, HFF-Vit-GV, M + HFF-Vit-GV, and C-Vit-GV oocytes.

**Conclusion:**

Employing an appropriate technique of vitrification followed by suitable IVM conditions can lead to reasonable IVF outcomes which may not benefit from extra supplementations. However, whether utilizing other supplementation formulas could improve the outcome requires further investigation.

## 1. Introduction

Obtaining qualified oocytes from ovarian stimulation procedures has remained a challenge in common assisted reproductive technologies (ART) (1).

Some harvested immature oocytes may not respond to in vivo gonadotropin stimulus and ART programs, but many are still capable of undergoing spontaneous in vitro maturation (IVM) and in vitro fertilization (IVF) in suitable culture conditions; therefore, the IVM technique was introduced (2). Cryopreservation of immature oocytes and IVM may preserve fecundity in some cases including ovarian cancer. Vitrification, which has the lowest rate of cryoinjuries, is considered the most appropriate method of cryopreservation in oocytes derived from humans and other species. Surveillance of human oocytes following vitrification, warming, and the IVM processes widely varies based on the exposure time, concentration of cryoprotectant solutions, rate of ice-crystal formation, and IVM protocol (3). Various problems are associated with the freezing of oocytes, including most commonly, spindle disorganization, microtubule disturbances, and increased risk of polyploidy at fertilization (4).

Follicular fluid (FF) contains various hormones, growth factors, glycosaminoglycans, and steroids (5). It is also rich in components crucial to oocyte maturation and embryo development including nutrients, gonadotropins, apoptosis inhibitors, meiosis-activating sterol, etc. IVM outcomes with autologous or heterologous FF are not consistent, and depend on the size of the follicles, the FF resource, and the concentration of FF components (6).

Melatonin is an indolamine derived from the amino acid tryptophan. The pineal gland is the main source of melatonin production, but it is also secreted in other parts of the body such as in the lacrimal gland, gastrointestinal tract, skin, ovary, testes, and liver. This indolamine is responsible for controlling the circadian rhythm and seasonal reproductive regulation; however, recent findings suggest melatonin also has an immunomodulatory function and cytoprotective role. As an antioxidant, melatonin induces DNA repair mechanisms, and regulates the action of other antioxidant agents and cell metabolism (7). Melatonin also plays a role in ovarian function and female reproduction. Supplementation of the culture medium with melatonin increases the rate of fertilization and enhances the earlier stages of embryonic development (8). Embryo culture medium supplemented with melatonin has improved blastocyst formation, the hatched blastocyst rate and the total blastomere number (9). In humans, melatonin could alter oxidative stress in FF and improve fertilization outcomes following oral consumption (10). Indolamine receptors have been detected in cumulus cells and oocytes (11), suggesting they have a role in oocyte development. Some in vitro studies have also demonstrated positive effects of melatonin use in oocyte maturation protocols and embryo culture. However, the use of melatonin in IVM may not affect (12), inhibit (13), or enhance nuclear maturation (11), and may also have no beneficial effect on embryo development (12).

Considering the controversial impact of FF and melatonin, the aim of the present study was to determine whether supplementation of IVM culture medium with a defined concentration of exogenous melatonin and FF could have any effect on nuclear and cytoplasmic maturation of vitrified oocytes.

## 2. Materials and Methods 

### Animals

Thirty 6-8-wk-old female NMRI mice were maintained in a 12-hr light/dark cycle at 22-24°C with unrestricted access to food and tap water.

### Chemicals 

All materials used in the study were purchased from Sigma Chemical Company (St Louis, MO, USA) unless stated otherwise.

### Study design and treatment interventions

This study was conducted between June 2017 and November 2018, at Afzalipour Medical School of Kerman University of Medical Sciences, Kerman, Iran. In vivo matured MII (Vivo-MII) oocytes were used as a control for the IVM conditions following ionomycin oocyte activation. The development of Vivo-MII oocytes to two-cell-stage embryos was compared with that of the in vitro matured GV oocytes (non-Vit-GV) group after ionomycin oocyte activation.

Vitrified GV (Vit-GV) oocytes were randomly assigned to control and treatment groups. The control group (C-Vit-GV) received no treatment, and its competence to undergo IVM and IVF was compared with the non-Vit-GV oocytes in order to estimate probable vitrification cryoinjuries.

The remaining vitrified-warmed oocytes were randomly allocated to the following groups: melatonin-treated Vit-GV (M-Vit-GV) oocytes, which were supplemented with 10 µM melatonin in the culture medium; HFF-treated Vit-GV (HFF-Vit-GV) oocytes, which were cultured in 40% human follicular fluid (HFF) in the culture medium; and melatonin + HFF treated Vit-GV (M + HFF-Vit-GV) oocytes, which were supplemented with 10 µM melatonin and 40% HFF. IVM and IVF competence of the different treated groups were compared with that of the C-Vit-GV group.

### HFF

Samples of FF were collected from women aged < 35 yr, with follicles > 14 mm diameter at day 11-12 of their menstrual cycle, and without polycystic ovaries. Aspirated FF containing mature oocytes was centrifuged at 300 × g for 10 min. The supernatant was collected in a new clean tube, inactivated at 56°C for 30 min, double filtered with 1.2 and 0.22 µm filters (Millipore Corporation, Bedford, MA), and stored at -20°C for further use.

### GV and MII oocyte collection 

In order to collect GV oocytes, the animals were sacrificed, and the ovaries were removed and immediately placed in an α-MEM culture medium. Cumulus-oocyte complexes (COCs) were harvested from antral follicles using a 28 G syringe needle. COCs were transferred into a pre-warmed culture medium and washed twice, then denuded by a 28 G needle and repeated pipetting, washed three times in the culture medium, and assessed for the presence of a GV, under an inverted microscope (Nikon TS100, Japan). The GV oocytes were relocated into drops of culture medium under mineral oil and incubated in a humid atmosphere of 5% CO ${}_{2}$ at 37°C. In order to collect MII oocytes, the female mice were superovulated by intraperitoneal injection of 10 IU of pregnant mare's serum gonadotropin, at 12-15 pm, followed by 10 IU of human chorionic gonadotropin (hCG, Serono, the Netherlands) 48 hr later. MII oocytes were collected from the fallopian tubes' ampulla 13-15 hr after the hCG treatment and transferred to drops of the medium under mineral oil.

### GV oocyte vitrification and warming

GV oocytes were exposed to an equilibration solution containing 20% human serum + 7.5% ethylene glycol (Merck, Germany) + 7.5% dimethyl sulfoxide (DMSO, Merck, Germany) in the culture medium for 10 min at room temperature, and a vitrification solution containing 20% human serum + 15% ethylene glycol + 15% DMSO + 0.5 M sucrose in the culture medium for 45 sec at room temperature.

The cryotop method was employed to transfer the oocytes to liquid nitrogen. Vitrified oocytes were removed from the liquid nitrogen, warmed rapidly, and transferred to an equilibration solution (α-MEM supplemented with 20% human serum and 1-mole sucrose) for 1 min. The vitrified oocytes were then transferred to a decreasing concentration of sucrose (1, 0.5, and 0.25-mole sucrose) for 3-5 min. Finally, the oocytes were washed three-five times in α-MEM with 20% human serum. The oocytes were examined under an inverted microscope and the viable oocytes were collected in drops of culture medium under mineral oil for further experiments.

### Oocyte viability assessment and IVM

After incubation of the oocytes for one hr in 5% CO ${}_{2}$ in humidified air at 37°C, the oocytes were assessed for viability and the ones with dark and highly granulated cytoplasm were excluded. The surviving oocytes were allocated to different treatment groups as noted in the study design, cultured in IVM medium containing α-MEM supplemented with 100 U FSH, 10 U hCG, 100 mL FBS, and 10 mL P/S per liter, and maintained for 48 hr in an atmosphere of 5% CO ${}_{2}$ in humidified air at 37°C. The oocytes were then observed every 24 hr by an inverted microscope to assess the oocyte maturity indicated by germinal vesicle breakdown (GVBD) and extrusion of the first polar body to the perivitelline space (MII oocytes).

### Artificial oocyte activation

In order to determine the effects of melatonin and HFF treatments on the fertilization capacity of in vitro matured MII oocytes, the GV oocytes were transferred to 10 µmol of ionomycin and 2 mmol of 6-dimethyl-aminopurine in α-MEM supplemented with 5% human serum for six min and three hr, respectively. The MII oocytes were then transferred to α-MEM with 20% human serum and assessed for the development to two-cell-stage embryos 24 and 48 hr later using an inverted phase-contrast microscope (Olympus, IX71, Japan).

### Ethical considerations

The experiments and animal use were approved by the Ethics Committee of Kerman University of Medical Sciences, Kerman, Iran (Code: IR.KMU.REC.1394.103) and were conducted according to the guidelines for the care and use of laboratory animals. A written informed consent was obtained from all women whose FF was used in the study.

### Statistical analysis

A Chi-square test was performed to compare the maturation and fertilization rates. Statistical analysis between the treatment groups was performed using Statistical Package for the Social Sciences software version 16.0 (SPSS Inc., Chicago, IL, USA). A p-value < 0.05 was considered significant.

## 3. Results

The outcomes related to the effect of melatonin, HFF, and their combination on IVM (development to GVBD and MII) and IVF (development to two-cell embryo) of 500 vitrified mouse oocytes are presented below.

### Competence of the IVM medium

**Figure 1:**
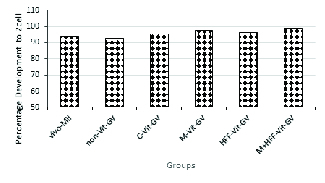
Development of in vivo and in vitro matured MII oocytes to 2 cell stage in the treatment and control groups 24 hr after Ionomycin and 6-dimethyl-aminopurin treatment. Vivo-MII: In vivo matured, Non-Vit-GV: Non-vitrified germinal vesicle, C-Vit-GV: Control for vitrified germinal vesicle, M-Vit-GV: Melatonin-treated vitrified germinal vesicles, HFF-Vit-GV: Human follicular fluid-treated vitrified germinal vesicle, M+HFF-Vit-GV: Melatonin+human follicular fluid-treated vitrified germinal vesicle.

The developmental rate of non-Vit-GV and Vivo-MII oocytes to two-cell embryos was compared to assess the competence of the IVM conditions. Following artificial oocyte activation, 94% of Vivo-MII oocytes and 93% of non-Vit-GV oocytes developed to two-cell stage embryos (Figure 1), so the results were comparable between the groups.

After one hr incubation of warmed Vit-GV oocytes in the culture medium, the oocytes were classified as alive oocytes (75.5%) with homogeneous clear cytoplasm and dead oocytes (24.5%) with dark and granulated cytoplasm; the latter oocytes were excluded. The competence of the IVM conditions was assessed by counting GVBD and MII oocytes in the non-Vit-GV and C-Vit-GV groups after 24 and 48 hr of IVM respectively.

74% of non-Vit-GV oocytes underwent GVBD/MII maturation after 24 hr (21% GVBD and 53% MII) and 88% of Vit-GV oocytes did so (17% GVBD and 71% MII). These values changed to 82% (12% GVBD and 70% MII) and 94% (8% GVBD and 86% MII), respectively, 48 hr after the incubation (Tables I). The difference was not statistically significant between the groups, indicating no deleterious effects of the vitrification protocol.

Table I presents the developmental competence of the 400 surviving oocytes distributed in the C-Vit-GV and three treatment groups (M-Vit-GV, HFF-Vit-GV, and M + HFF-Vit-GV) after 24 and 48 hr of IVM. No significant difference was detected among the groups.

### Oocyte activation and development to two-cell embryos

Observing the pronucleus in any of the matured oocytes (MII) was considered the initial sign of oocyte activation to undergo development to the two-cell stage. Data shown in Figure 1 suggest that (i) in vivo matured oocytes underwent the two-cell stage at a rate comparable with the non-vitrified in vitro matured GV oocytes (non-Vit-GV); (ii) vitrified in vitro matured GV oocytes (C-Vit-GV) underwent two-cell stage at a comparable rate as the non-Vit-GV oocytes; and (iii) in-vitro development of vitrified in vitro matured oocytes following artificial oocyte activation by ionomycin was higher when the culture media were enriched by melatonin, HFF and especially their combination. However, none of the treatments resulted in a significant difference compared with the C-Vit-GV group of oocytes.

**Table 1:** Effect of melatonin and HFF on in vitro maturation of mouse vitrified oocytes after 24 and 48 hr of culture


	**Maturation at 24 hr**					**Maturation at 48 hr**				
	**GV**	**GVBD**	**MII**	**GVBD + MII**	**DEG**	**GV**	**GVBD**	**MII**	**GVBD + MII**	**DEG**
**Non-Vit-GV**	15	21	53	74	11	7	12	70	82	11
**C-Vit-GV**	7	17	71	88	5	1	8	86	94	5
**M-Vit-GV**	11	19	63	82	7	1	10	82	92	7
**HFF-Vit-GV**	10	16	68	84	6	4	6	84	90	6
**M + HFF-Vit-GV**	14	20	60	80	6	6	12	76	88	6
Data presented as percentage development to the anticipated stage, GV: Germinal vesicle, GVBD: Germinal vesicle breakdown, MII: Metaphase 2, DEG: Degenerated, Non-Vit-GV: Non-vitrified germinal vesicle, C-Vit-GV: Control for vitrified germinal vesicle, M-Vit-GV: Melatonin-treated vitrified germinal vesicles, HFF-Vit-GV: Human follicular fluid-treated vitrified germinal vesicle, M + HFF-Vit-GV: Melatonin + human follicular fluid-treated vitrified germinal vesicle										

## 4. Discussion

In the present study, vitrified mouse GV oocytes underwent IVM and their viability and maturation were assessed to determine whether the vitrification process had any harmful effects. We found that vitrified GV oocytes could reach a maturation rate comparable with that of non-vitrified oocytes and therefore vitrification had no negative impact on the maturation of GV oocytes, evaluated 24 and 48 hr post warming. Theoretically, GV oocytes, due to the presence of a nuclear membrane and diffused chromatins, are more resistant to cryoinjuries and chromosomal disorders (3); however, the maturation potential of oocytes aspirated from stimulated ovaries following cryopreservation is controversial due to factors such as the presence of cumulus cells, culturing duration, oocyte maturation level, and IVM media. A decrease in the maturation and viability rate of vitrified human oocysts was reported when compared with non-vitrified human oocytes (14). However, no differences in the incidence of diploid metaphase II bovine oocytes among control, vitrified, and non-vitrified groups has been detected (15). Similar survival rate in both mature and immature vitrified oocytes after warming was also reported. However, they found a significantly higher oocyte maturation rate in the oocytes that had undergone IVM before vitrification than in the oocytes vitrified after IVM (16). Our results also revealed that the employed IVM protocol could support the maturation of vitrified GV oocytes as no significant difference was found regarding the competence to undergo GVBD and MII stage.

FF has a crucial role in nutritional and hormonal support of oocytes particularly before ovulation (17). However, findings from other studies regarding the positive impact of FF on in vitro conditions are inconsistent. When GV oocytes were cultured in pure FF derived from small follicles 1-5 mm in diameter, the maturation rate was significantly low (18). In contrast, a higher rate of pronucleus formation was detected when the culture medium was enriched with porcine FF (19). Other studies have also reported that supplementation of maturation medium with 10% FF obtained from small, medium, large, and pre-ovulatory follicles (18), and also 20% FF (20) improved the maturation potential of bovine oocytes. In the present study, addition of FF to the oocyte maturation medium resulted in a slight difference in the maturation and fertilization rate between the C-Vit-GV and HFF-Vit-GV groups. The low inhibitory effect of FF in our experiment could be due to the addition of FSH which is known to be a functional blocker of oocyte maturation inhibitors and also meiosis arrest substances in FF, such as coagulants and inhibitory and fibrinolytic factors. Also, the absence of related promotional effects of FF on the maturation of oocytes and embryo development could be explained by protein denaturation and formation of toxic levels of ammonia in the culture medium. Of note, none of the aforementioned studies addressed the effects of active species of oxygen on the maturity potential of GV oocytes.

Some studies have demonstrated the beneficial effects of ascorbate, tocopherol, and melatonin as the most well-known antioxidants that may aid in oocyte recovery after vitrification (21, 22). Melatonin has been used in egg, sperm and embryo vitrification procedures to assist with metabolic regulation and ROS level control (23), which has led to decreased two-cell block, apoptosis and embryonic fragmentation, the promotion of sex steroid hormone production, facilitation of nuclear and cytoplasmic maturation, and enhancement of the blastulation rate, blastocyst cell numbers, and embryo implementation (8). Although the majority of reports claim the beneficial effect of melatonin administration in post warming procedures of IVM and IVF of oocytes at various stages of maturation (24), evidence suggests that melatonin effectiveness widely varies based on the experimental model, exposure duration, supplementation dose, indolamine origin, and maturation stage of the oocyte (25).

We observed that treatment of IVM and IVF media with melatonin seemed to have largely unaffected the vitrified GV oocytes in their completion of meiosis and embryo development, as there was an insignificant difference in MII and two-cell parameters between the treatment and control groups. This phenomenon might have been a result of vitrification prior to maturation, as GV oocytes are less sensitive to vitrification side effects compared with MII ones, even in the case of melatonin supplementation. It has been shown that early-stage embryos have little response to melatonin supplementation; accordingly, Bahadori and colleagues in 2013 observed decreased melatonin receptor expression in the mouse embryos that they studied (26). On the other hand, the dose-dependent effect of melatonin on vitrified MII oocytes revealed that not only did the addition of melatonin at 10 ${}^{-9}$ M and higher concentrations result in significant beneficial effects, but also it was cytotoxic at 10 ${}^{-3}$ M (27). In our study, neither the recommended dose of melatonin (10 ${}^{-5}$ M) (8) nor 40% HFF, nor their combination, significantly improved the maturation and developmental rate of vitrified GV oocytes. As the maturation rate of in vivo matured and non-vitrified GV oocytes were comparable with the vitrified oocytes, it can be deduced that the capability of the vitrification procedure and the maturation and fertilization media were adequate to support the procedure.

To the best of the authors' knowledge, the present study is the first to investigate the effect of HFF and melatonin separately and in combination on vitrified mouse immature oocytes and on different maturation stages continuously in the same media. We used the ionomycin oocyte activation protocol which is more consistent than sperm-mediated oocyte activation, in which the results are prone to variations in the sperm quality. However, supplementing media with melatonin, HFF, and their combination through a prolonged culture may lead to different outcomes of embryo development, which cannot be examined from our study and so warrants further investigation.

## 5. Conclusion

It can be concluded that GV oocyte vitrification is a promising method of cryopreservation that can be used for patients with ovarian failure and other related health conditions. When maturation and fertilization media are efficient, supplementation of culture media with enhancing factors such as FF, melatonin, etc. could exert little/if any positive impact on IVM/IVF outcomes in vitrified GV oocytes. However, further studies are required to identify the vitrified GV oocytes' needs and the extent to which different culture supplements can influence ART outcomes.

##  Conflict of Interest

The authors declare that they have no conflict of interest.
